# Sensitivity and Specificity of Bacterial Vaginosis Rapid Test Kit in Diagnosing Bacterial Vaginosis: A Prospective Observational Study

**DOI:** 10.7759/cureus.104963

**Published:** 2026-03-10

**Authors:** Katta Sushma, Aruna Biradar, Neelamma Patil, Shobha Shiragur, Ekta Chhabra, Santosh Arakeri, Jyoti Lokapur, Apoorva Tippabhotla, Anuradha Khavekar, Ashok Reddy

**Affiliations:** 1 Obstetrics and Gynaecology, Shri B.M. Patil Medical College, Hospital and Research Centre, Bharatiya Lingayat Development Educational Association (BLDE) (Deemed to be University), Vijayapura, IND

**Keywords:** bacterial vaginosis, bv rapid test kit, diagnostic accuracy, pap smear, vaginal ph

## Abstract

Introduction: Bacterial vaginosis (BV) is the most common cause of abnormal vaginal discharge among women of reproductive age and results from disruption of the normal vaginal microbiota. It is associated with adverse gynecological and obstetric outcomes, underscoring the need for early and accurate diagnosis. Conventional diagnostic methods require microscopy and laboratory expertise, limiting their applicability in resource-constrained settings. The present study aimed to evaluate the sensitivity and specificity of the BV rapid test kit, primarily based on vaginal pH detection, in diagnosing bacterial vaginosis among symptomatic women.

Materials and methods: This prospective observational study was conducted over 18 months from June 2024 to December 2025 at Shri B.M. Patil Medical College Hospital and Research Centre, Vijayapura. A total of 285 married women aged 18-45 years presenting with complaints of vaginal discharge were included. Vaginal swabs were collected for the BV rapid test kit analysis and Papanicolaou (Pap) smear examination. Vaginal pH was measured using the test kit, and a pH value ≥4.6 was interpreted as suggestive of bacterial vaginosis according to the manufacturer’s protocol. Pap smear findings were considered the reference standard for evaluating diagnostic performance. Sensitivity, specificity, positive predictive value, negative predictive value, and overall diagnostic accuracy were calculated.

Results: Out of 285 married women, the mean age of the participants was 34.78 ± 6.66 years. Pap smear examination revealed infective pathology in 115 (40.4%) women, including bacterial vaginosis in 85 (29.8%) and candidiasis in 30 (10.5%). Elevated vaginal pH (≥4.6) was observed in 114 (40.0%) participants and demonstrated a statistically significant association with infective pathology (p < 0.001). The BV rapid test kit showed a sensitivity of 73.91% and a specificity of 82.94%. The positive predictive value and negative predictive value were 74.56% and 82.46%, respectively, with an overall diagnostic accuracy of 79.30%.

Conclusion: The BV rapid test kit demonstrated moderate sensitivity and good specificity in detecting bacterial vaginosis among symptomatic women. Its rapid turnaround time, ease of use, and minimal infrastructure requirements make it a practical adjunct screening tool, particularly in settings where microscopy-based methods are not readily available. Incorporating point-of-care testing may facilitate timely treatment initiation and potentially reduce complications associated with untreated bacterial vaginosis.

## Introduction

Bacterial vaginosis (BV) is the most prevalent cause of abnormal vaginal discharge among women of reproductive age and is usually brought on by an imbalance of the normal vaginal flora, characterized by an increase in anaerobic bacteria and a decrease in lactobacilli [1]. According to the Amsel criteria, BV is diagnosed by the presence of at least three of the following four features: homogeneous vaginal discharge (thin, homogeneous, grey or white discharge); positive whiff test (characterized by a fishy odor on addition of 10% potassium hydroxide); high vaginal pH (>4.5); and identification of vaginal epithelial cells heavily coated with bacteria (clue cells) [2].

Bacterial vaginosis has significant adverse effects on the quality of life of women, particularly affecting sexual, physical, mental, and social health [3]. More importantly, BV is associated with several adverse obstetric and gynecological outcomes, and it has been linked to preterm birth, preterm premature rupture of membranes (PPROM), chorioamnionitis, postpartum endometritis, post-abortal endometritis, and an increased risk of miscarriage, particularly in women undergoing assisted reproductive techniques (ART) [4]. Therefore, the importance of early and accurate diagnosis cannot be overemphasized.

The Amsel criteria and Nugent scoring system are two of the most commonly used methods for the diagnosis of BV [2,5,6]. Both methods involve microscopic examination of vaginal samples. However, these tests require skilled personnel and laboratory infrastructure and are relatively time-consuming, which limits their utility in resource-limited settings [1]. Although the Amsel criteria are widely used in clinical practice, their sensitivity is generally lower than that of the Nugent scoring system, and the diagnostic accuracy may vary between observers [6].

In addition to the Amsel criteria, several point-of-care tests are available for the diagnosis of BV [1]. One such test is the BV Blue test, which is a rapid diagnostic method based on the detection of elevated levels of sialidase enzyme activity in vaginal fluid samples [1]. The detection and quantification of microbial enzymes, particularly sialidases, have shown promise in the rapid diagnosis of BV [1,7]. Apart from enzymatic detection methods, point-of-care diagnostic kits based on vaginal pH estimation are also increasingly used in outpatient settings because of their simplicity, rapid turnaround time, and minimal requirement for laboratory infrastructure [1,7].

A single unit of sialidase activity is defined as the amount of enzyme required to release 1 nmol of substrate per milliliter per minute at 37°C [1,8]. The BV Blue test involves collecting vaginal secretions using a cotton swab, which is then placed in a test tube containing sialidase-binding substrate, and the tube is incubated at room temperature for 10 minutes [1]. Subsequently, one or two drops of chromogenic reagent are added, and the mixture is further incubated for 3 minutes. A blue or green color indicates a positive result, whereas a yellow color indicates a negative result [1,8].

The major strength of this test lies in its rapidity and simplicity. However, it has certain limitations, such as it may fail to detect bacterial species that do not produce sialidase enzymes, despite being associated with BV, and the presence of other sexually transmitted infections (STIs), such as Trichomonas vaginalis, or coexisting vaginal candidiasis may influence the test results, and also, similar to the Amsel criteria, it does not assess the severity of BV [1,8]. Similarly, pH-based rapid tests may be influenced by physiological factors such as hormonal variations or vaginal atrophy, which may affect test specificity in certain populations.

In this context, the present study aimed to evaluate the diagnostic performance of a point-of-care BV rapid test kit primarily based on vaginal pH detection and to determine its sensitivity and specificity in the diagnosis of bacterial vaginosis among symptomatic women.

## Materials and methods

This prospective observational study was conducted from June 2024 to December 2025 in the Department of Obstetrics and Gynecology at Shri B.M. Patil Medical College Hospital and Research Centre, Vijayapura, over a period of 18 months. Ethical clearance was obtained from the Institutional Ethics Committee (IEC Number: SBMPMC/116/2023-24) prior to the commencement of the study. All participants were enrolled only after obtaining written informed consent.

The study population consisted of married women aged between 18 years and 45 years who attended the outpatient department with complaints of white vaginal discharge. Married women were included because, in the local sociocultural context, sexually active women attending the gynecology outpatient department are predominantly married, which facilitated the reliable collection of reproductive and sexual health history. Women presenting with vaginal bleeding along with discharge, those who had used systemic or topical antibiotics, antifungal medications, or over-the-counter vaginal preparations within the preceding two weeks, and those who declined participation were excluded from the study. Participants meeting the eligibility criteria were recruited consecutively until the desired sample size was achieved.

The required sample size was calculated based on the previously reported sensitivity (52%) and specificity (95%) of Amsel’s criteria among symptomatic women, with an assumed disease prevalence of 34%, 95% confidence level, and desired precision of 1% [9]. The sample size formula used for this diagnostic accuracy study was:

N = (a + c) / Prevalence,

where “a” and “c” represent the expected number of true positives and false negatives used to approximate sensitivity within the target population. Using the above formulae, the minimum sample size required was determined to be 285 participants.

A detailed clinical history was obtained from each participant, including obstetric history, menstrual history, contraceptive use, sexual history, and prior treatment for vaginal infections. This was followed by a general physical examination and systemic examination. A thorough gynecological examination was performed under aseptic precautions. A per speculum examination was carried out to assess the nature of vaginal discharge and to facilitate specimen collection. Vaginal swabs were obtained from the lateral vaginal wall and posterior fornix, which are considered appropriate sites for microbiological evaluation in cases of suspected bacterial vaginosis [10].

Two separate high vaginal swabs were collected from each participant. The first swab was used for the BV rapid test kit, and the second swab was utilized for the Pap smear examination. For the BV rapid test, the vaginal swab was applied to the designated test area of the kit according to the manufacturer’s instructions. After approximately 20 seconds, the color change was observed under adequate lighting conditions and compared with the reference color chart provided with the kit. Vaginal pH was assessed using the rapid test kit, and a pH value ≥4.6 was considered suggestive of bacterial vaginosis according to the manufacturer’s interpretation protocol. Although traditional Amsel criteria consider vaginal pH >4.5, the present study adhered to the kit-specified threshold to ensure standardized interpretation of results.

The Pap smear was prepared using conventional cytology techniques and evaluated according to the Bethesda System for Reporting Cervical Cytology [11]. Nugent scoring using Gram staining, considered the gold standard for bacterial vaginosis diagnosis, was not routinely available in our institutional setting during the study period; therefore, Pap smear cytology was used as an accessible objective reference method for evaluating diagnostic performance.

All clinical findings and laboratory results were recorded in a structured pro forma designed for the study. The collected data was entered into Microsoft Excel and analyzed using the IBM Corp. Released 2020. IBM SPSS Statistics for Windows, Version 26. Armonk, NY: IBM Corp. Categorical variables were presented as frequency and percentage and analyzed using the chi-square test. Diagnostic accuracy parameters, including sensitivity, specificity, positive predictive value (PPV), and negative predictive value (NPV), were calculated to assess the performance of the BV rapid test kit. A p-value of less than 0.05 was considered statistically significant.

## Results

**Figure 1:**
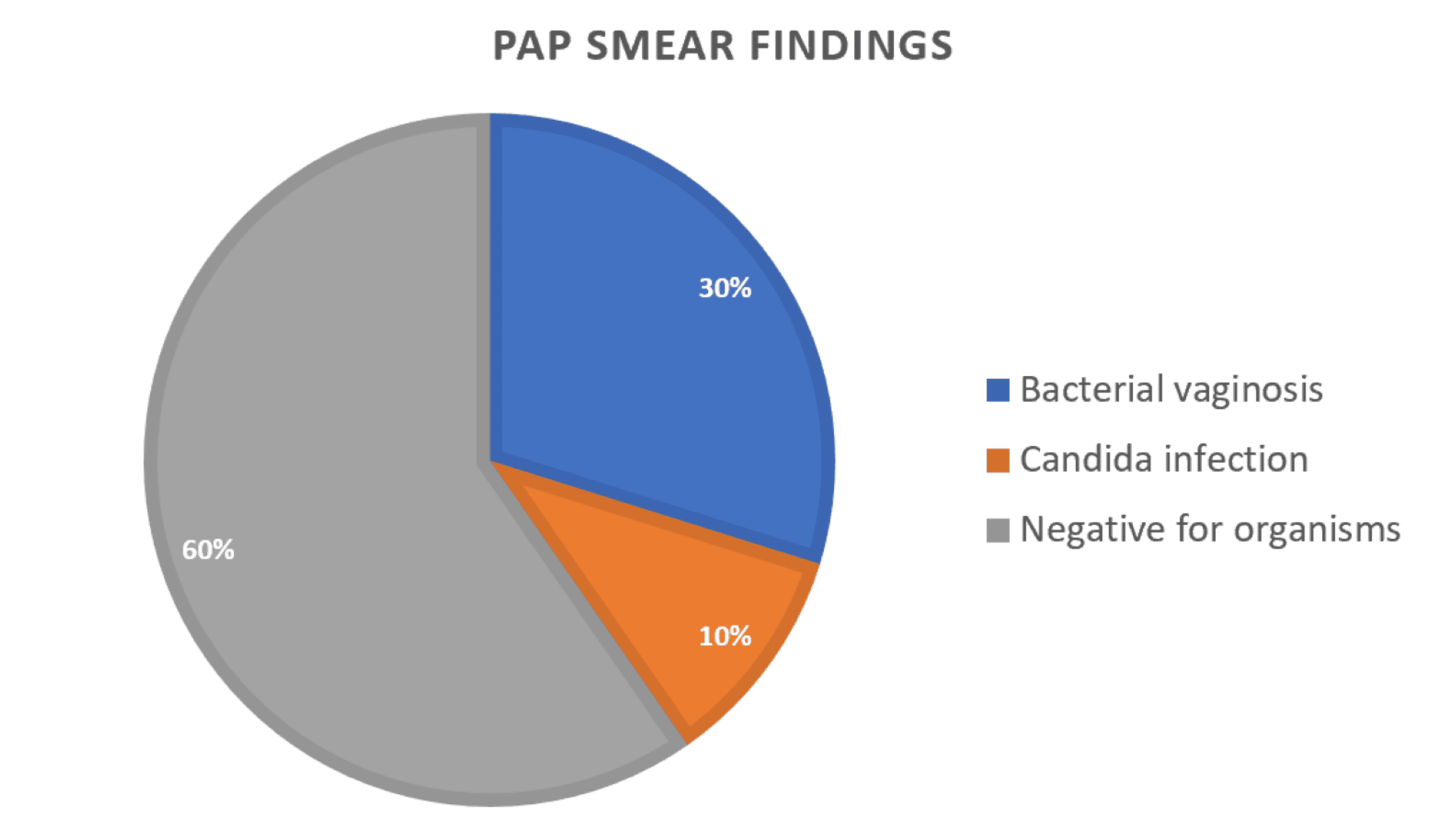
Pie Chart Showing Distribution of Pap Smear Examination Findings

Out of 285 married women, the mean age of the study participants was 34.78 ± 6.66 years, and the majority of participants were aged >41 years (75, or 26.3%). Pap smear examination revealed that bacterial vaginosis was detected in 85 (29.8%) women, while Candida infection was identified in 30 (10.5%) women. The majority of participants, 170 (59.7%), were negative for infective organisms. Overall, infective pathology was observed in 115 (40.4%) women, indicating that nearly two-fifths of the study population had microbiological evidence of vaginal infection on cytology (Table 1) (Figure 1).

**Table 1:** Distribution of Pap Smear Examination Findings (n = 285) This table presents the distribution of Pap smear examination findings among 285 study participants. The table includes the number of patients (n) and percentage (%) for bacterial vaginosis, Candida infection, and cases negative for organisms. The total sample size (n = 285) and corresponding percentages are provided. n: number of patients; %: percentage.

Pap smear findings	Number of patients (n)	Percentage (%)
Bacterial vaginosis	85	29.8
Candida infection	30	10.5
Negative for organisms	170	59.7
Total	285	100.0

Among women with vaginal pH ≥4.6, 85 (73.9%) had positive Pap smear findings, whereas only 30 (26.1%) women with pH ≤4.5 showed evidence of infection. Conversely, Pap smear negativity was significantly higher in women with pH ≤4.5 (141 [82.9%]) compared to those with pH ≥4.6 (29 [17.1%]). Elevated vaginal pH (≥4.6) was observed in 114 (40.0%) participants and demonstrated a strong association with infective pathology (chi-square = 92.3; p < 0.001), indicating a statistically significant correlation between increased vaginal pH and the presence of vaginal infection (Table 2).

**Table 2:** Association Between Vaginal pH (BV Rapid Test Kit) and Pap Smear Findings (n = 285) This table presents the cross-tabulation between vaginal pH levels measured using the BV rapid test kit and Pap smear findings (positive or negative for organisms) among 285 participants. Data are expressed as numbers (percentages) in each category. The total number and percentage distribution are shown. The Chi-square test was used to assess the association between categorical variables. The chi-square value was calculated as 92.3; and the p-value was calculated as < 0.001. Statistical test used: Pearson’s Chi-square test. BV: bacterial vaginosis; n: number of patients; %: percentage; p: probability value.

Vaginal pH (BV kit)	Pap smear positive for organisms	Pap smear negative for organisms	Total n (%)
≥ 4.6	85 (73.9%)	29 (17.1%)	114 (40.0%)
≤ 4.5	30 (26.1%)	141 (82.9%)	171 (60.0%)
Total	115 (100%)	170 (100%)	285 (100%)

The BV rapid test kit demonstrated a sensitivity of 73.91% (95% CI: 64.90-81.66) and a specificity of 82.94% (95% CI: 76.43-88.27). The positive predictive value was 74.56% (95% CI: 67.40-80.60), and the negative predictive value was 82.46% (95% CI: 77.42-86.56). The overall diagnostic accuracy was 79.30% (95% CI: 74.12-83.85), with a disease prevalence of 40.35% (95% CI: 34.61-46.30). These findings indicate that the BV rapid test kit has good specificity and moderate sensitivity for the detection of bacterial vaginosis in symptomatic women (Table 3).

**Table 3:** Diagnostic Performance of BV Rapid Test Kit (n = 285) This table presents the diagnostic performance parameters of the BV rapid test kit in comparison with Pap smear findings among 285 participants. The parameters included are sensitivity, specificity, disease prevalence, positive predictive value, negative predictive value, and diagnostic accuracy. Values are expressed as percentages along with their corresponding 95% confidence intervals. BV: bacterial vaginosis; CI: confidence interval; %: percentage.

Diagnostic Parameter	Value (%)	95% Confidence Interval
Sensitivity	73.91	64.90 – 81.66
Specificity	82.94	76.43 – 88.27
Disease prevalence	40.35	34.61 – 46.30
Positive Predictive Value	74.56	67.40 – 80.60
Negative Predictive Value	82.46	77.42 – 86.56
Diagnostic Accuracy	79.30	74.12 – 83.85

## Discussion

In the present study, a higher proportion of women belonged to the late reproductive and perimenopausal age groups, with the largest representation among women aged >41 years (75, 26.3%), followed by those aged 36-40 years (64, 22.5%). Similar age-related trends have been reported by Madhivanan P et al. and Farheen A et al., who observed increased BV prevalence among women above 36 years of age [9,12]. These findings may be attributed to hormonal fluctuations, alterations in the vaginal microenvironment, and behavioral factors influencing the vaginal flora. It is also important to note that vaginal pH tends to increase with declining estrogen levels during the perimenopausal period. Therefore, elevated vaginal pH in women above 40 years of age may partly reflect physiological hormonal changes rather than infection alone, which could potentially influence the specificity of pH-based diagnostic tests. Although vaginal discharge was the predominant complaint in all 285 (100%) participants, associated symptoms such as burning micturition, pruritus, foul-smelling discharge, and lower abdominal pain were also reported. Comparable symptom profiles were described by Farheen A. et al. and Khedkar R. et al. [12,13]. However, as highlighted by Klebanoff et al., symptoms alone are unreliable predictors of BV, as similar clinical features may be present in women without microbiological confirmation [14]. This emphasizes the need for objective diagnostic evaluation.

Pap smear examination demonstrated infective pathology in 115 (40.4%) women, with bacterial vaginosis identified in 85 (29.8%) and candidiasis in 30 (10.5%). The remaining 170 (59.7%) women were negative for organisms. A comparable proportion of BV detection on cytology was reported by Anand et al. [15]. Although primarily utilized for cervical cancer screening, the Pap smear can provide indirect evidence of alterations in vaginal flora. Previous studies have shown a correlation between coccobacillary predominance on cytology and Gram stain-confirmed BV [16]. Nevertheless, its sensitivity for diagnosing BV remains variable. Tokyol C et al. reported relatively low sensitivity but high specificity, possibly due to the cervical origin of samples rather than direct vaginal sampling [17]. In many resource-limited settings where Gram staining and Nugent scoring are not routinely available, Pap smear cytology may serve as a pragmatic surrogate indicator of altered vaginal flora and has been utilized in several observational studies. Thus, while Pap smear findings may suggest infective changes, they may not reliably serve as a definitive diagnostic modality for BV.

Assessment of vaginal pH revealed that 114 (40.0%) women had a pH ≥4.6, whereas 171 (60.0%) had a pH ≤4.5. Among women with elevated vaginal pH (≥4.6), 85 (73.9%) had positive Pap smear findings, compared to 30 (26.1%) among those with pH ≤4.5. Conversely, Pap smear negativity was significantly higher among women with pH ≤4.5 (141 [82.9%]) compared to those with pH ≥4.6 (29 [17.1%]). Elevated vaginal pH demonstrated a strong and statistically significant association with infective pathology (χ² = 92.388, p = 0.001). Similar findings were reported by Shen CJ et al., who observed a significant correlation between elevated vaginal pH and clinically diagnosed vaginal infection [18]. These results support the utility of vaginal pH estimation as a simple and effective screening parameter in symptomatic women.

The BV rapid test kit demonstrated a sensitivity of 73.91%, a specificity of 82.94%, and an overall diagnostic accuracy of 79.30%, with disease prevalence observed in 115 (40.35%) women. The positive predictive value was 74.56%, while the negative predictive value was 82.46%, indicating that a negative test result reliably excluded infective pathology in a substantial proportion of cases. Comparable diagnostic performance was reported by Shen CJ et al., who documented a sensitivity of 86.7% and a specificity of 88.6% [18]. Foessleitner et al. observed high sensitivity (81%) and specificity (100%) when compared with Nugent scoring, particularly among asymptomatic pregnant women [19]. Bradshaw et al. and Myziuk et al. similarly demonstrated high diagnostic performance of the BV Blue test against Amsel criteria and Nugent scoring [8,20]. Conversely, Madhivanan et al. reported lower sensitivity in an Indian population, attributing this to differences in vaginal flora composition and the presence of sialidase-negative organisms [9]. Lokken et al. also emphasized population-specific variability in test performance [21]. Since sialidase activity is present in approximately 75-84% of BV cases, false-negative results may occur in settings where sialidase-negative strains are prevalent, which may partly explain the moderate sensitivity observed in the present study.

The study has certain limitations. A Pap smear was used as the reference standard, although it is not the gold standard for diagnosing bacterial vaginosis, which may have affected diagnostic accuracy estimates. The use of a Pap smear as a surrogate reference method was necessitated by the limited availability of Gram staining and Nugent scoring in the study setting. Being a single-center study, the findings may not be generalizable to other populations with different vaginal microbiota profiles. Additionally, only symptomatic married women aged 18-45 years were included, which may introduce selection bias and limit extrapolation of the findings to unmarried or asymptomatic women. Molecular diagnostic methods were not employed for detailed microbiological confirmation. Furthermore, clinical history regarding symptoms and prior treatment was obtained through patient self-report, which may be subject to recall bias.

## Conclusions

The present study suggests that vaginal pH testing using a BV rapid test kit demonstrates satisfactory diagnostic performance in the identification of bacterial vaginosis among symptomatic women. With moderate sensitivity and good specificity, the test provides a useful screening method for detecting infectious pathology in routine clinical settings. Although variations in diagnostic performance may occur across different populations due to differences in vaginal microbiota composition, the BV rapid test kit represents a practical, cost-effective, and rapid diagnostic modality.

Its ease of use, minimal requirement for laboratory infrastructure, and rapid turnaround time make it particularly valuable in resource-constrained settings where microscopy-based diagnostic methods may not be readily accessible. Incorporation of such point-of-care testing into routine gynecological practice may facilitate early diagnosis, prompt initiation of appropriate therapy, and reduce complications associated with untreated bacterial vaginosis. However, given the moderate sensitivity and potential for false-negative results, the test should preferably be used as an adjunct screening tool rather than a standalone diagnostic method, especially in cases with equivocal clinical findings. Further multicentric studies involving diverse populations and comparison with gold-standard diagnostic techniques may help to validate and strengthen its clinical applicability.
